# Interactions between the Intrinsically Disordered Regions of hnRNP-A2 and TDP-43 Accelerate TDP-43′s Conformational Transition

**DOI:** 10.3390/ijms21165930

**Published:** 2020-08-18

**Authors:** Wan-Chin Chiang, Ming-Hsuan Lee, Tsai-Chen Chen, Jie-rong Huang

**Affiliations:** 1 Institute of Biochemistry and Molecular Biology, National Yang-Ming University, No. 155 Section 2, Li-Nong Street, Taipei 11221, Taiwan; cmiyc777@gmail.com (W.-C.C.); lkm551200@gmail.com (M.-H.L.); hazelnut.chen.scu@gmail.com (T.-C.C.); 2 Institute of Biomedical Informatics, National Yang-Ming University, No. 155 Section 2, Li-Nong Street, Taipei 11221, Taiwan; 3 Department of Life Sciences and Institute of Genome Sciences, National Yang-Ming University, No. 155 Section 2, Li-Nong Street, Taipei 11221, Taiwan

**Keywords:** intrinsically disordered protein, liquid–liquid phase separation, membraneless organelle, NMR spectroscopy

## Abstract

Most biological functions involve protein–protein interactions. Our understanding of these interactions is based mainly on those of structured proteins, because encounters between intrinsically disordered proteins (IDPs) or proteins with intrinsically disordered regions (IDRs) are much less studied, regardless of the fact that more than half eukaryotic proteins contain IDRs. RNA-binding proteins (RBPs) are a large family whose members almost all have IDRs in addition to RNA binding domains. These IDRs, having low sequence similarity, interact, but structural details on these interactions are still lacking. Here, using the IDRs of two RBPs (hnRNA-A2 and TDP-43) as a model, we demonstrate that the rate at which TDP-43′s IDR undergoes the neurodegenerative disease related α-helix-to-β-sheet transition increases in relation to the amount of hnRNP-A2′s IDR that is present. There are more than 1500 RBPs in human cells and most of them have IDRs. RBPs often join the same complexes to regulate genes. In addition to the structured RNA-recognition motifs, our study demonstrates a general mechanism through which RBPs may regulate each other’s functions through their IDRs.

## 1. Introduction

Protein-protein interactions mediate the functions of life [1]. The atomic details of the quaternary structure of interacting proteins, obtained by X-ray crystallography [2], NMR spectroscopy [3,4], or cryogenic electron microscopy (cryo-EM) [5,6,7,8], provide snapshots of how they work. Although most of the binding events reported to date involve folded domains, interactions between or with intrinsically disordered regions (IDRs, present in more than half of eukaryotic proteins [9,10]) are also important in many biological reactions: An IDR can contact its folded partner by folding upon binding [11,12]; current literature suggests that it is also common for IDRs to bind “fuzzily” to their folded partners without adapting a fixed conformation [13,14,15]. There have only been a few reports on how two IDRs from different proteins interact [16,17,18]. This gap in our understanding of protein-protein interaction networks can be filled by discovering new interaction modes between IDRs.

**Figure 1:**
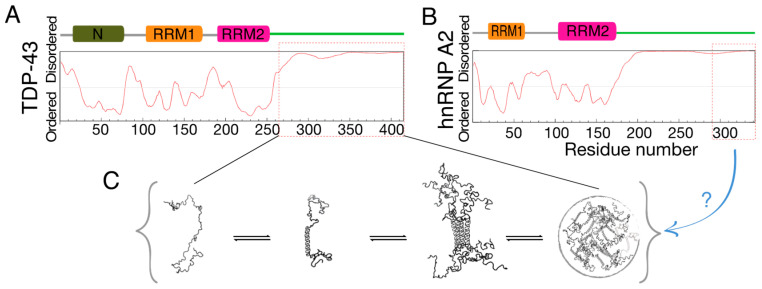
Schematic illustration of the intrinsically disordered regions (IDRs) of TDP-43 and hnRNP-A2 in this study. (**A**,**B**) The domains of (**A**) TDP-43 and (**B**) hnRNP-A2. The level of protein disorder was predicted using the PONDR VSL2 algorithm [36]. The fragments used in this study are indicated with red-dashed lines. (**C**) Schematic representation of the α-helical, self-association, and liquid-liquid phase separation propensities of TDP-43 reported in previous publications [37,38,39]. The molecular details of the effects of hnRNP-A2 remain elusive.

Almost all RNA-binding proteins (RBPs) have IDRs [19,20,21]. While RBPs are at the core of post-transcriptional gene regulation, the roles of their IDRs are only now coming to light. Studies show that some IDRs drive the location of the entire protein into biomolecular condensates (stress or RNA granules) via liquid–liquid phase separation (LLPS) [22,23,24,25,26] and that misregulation of condensate formation is pathogenic [27,28]. The IDRs of RBPs have also been shown in a few cases to interact with the IDRs of other proteins [29,30,31]. Overall, however, the roles of IDRs in this large (1500+ members [32]) protein family remain largely unexplored. Here, we demonstrate a new interaction mode, whereby the IDR of one RBP accelerates the aggregation of another RBP’s IDR. Although the interactions between the IDRs of TDP-43 and hnRNP-A2 and their functional importance have been reported previously [33,34,35], molecular details of the effects of the two IDRs on each other have remained elusive (Figure 1C). We show here that the amount of hnRNPA2 determines the rates of α-to-β structural transition of TDP-43.

## 2. Results and Discussion

**Figure 2:**
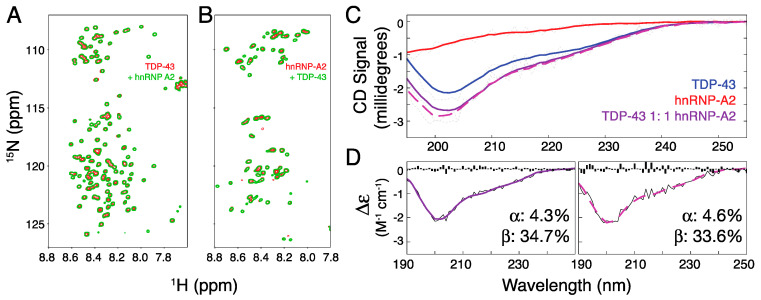
The interaction between the intrinsically disordered regions (IDRs) of TDP-43 and hnRNP-A2. (**A**) Overlaid NMR HSQC spectra of 20 µM ^15^N TDP-43^266–414^ alone (red) and in the presence of 20 µM ^14^N-hnRNP-A2^288–341^ (green). (**B**) Overlaid NMR HSQC spectra of 20 µM ^15^N- hnRNP-A2^288–341^ alone (red) and in the presence of 20 µM ^14^N-TDP-43^266–414^ (green). (**C**) Circular dichroism spectra of 20 µM TDP-43^266–414^ (blue), hnRNP-A2^288–341^ (red), and a one-to-one mixture of the two (purple) overlaid on the summed spectra of the two proteins alone (broken purple line). (**D**) Fits using BeStSel [41,42] of the mixture (left panel) and numerically summed TDP-43/hnRNP-A2 spectra (right) and the proportion of secondary structure components obtained. The residuals of the fits are shown as black bars.

TDP-43′s intrinsically disordered C-terminal domain is responsible for RNA splicing inhibition, and this function is reduced by its interaction with hnRNP-A2 [33]. The regions of the two proteins involved in their interaction were identified by co-immunoprecipitation, specifically, the interaction was confirmed by observing a disruption of the supershift in electrophoretic mobility shift assays of synthesized peptides corresponding to specific regions of the two proteins [34,35]. Although minimum fragments of residues 321–366 from TDP-43 and residues 288–341 from hnRNP A2 were identified, there was no evidence of direct interactions (e.g., from GST pull-down assays), indicating that the binding is probably weak and transient. This is consistent with evidence of a weak interaction from intermolecular paramagnetic relaxation enhancement NMR measurements (which probe transient interactions) [40]. In the present study, no significant changes were observed in the chemical shifts of the 20 µM ^15^N-labeled IDR of TDP-43 (TDP-43^266–414^; NMR-active) when it was mixed with a 20 µM minimum fragment of ^14^N hnRNP-A2 (hnRNP-A2^288–341^; NMR-inactive) and vice versa (Figure 2A,B). The fact that the summed circular dichroism (CD) spectra of hnRNP-A2^288–341^ and TDP-43^266–414^ (both 20 µM) alone overlap closely with the spectrum of the mixed sample (Figure 2C), indicates that there are no changes in secondary structure upon mixing. Quantitative analysis of the experimental data (Figure 2D, left) and the summed CD curves (Figure 2D, right) yielded similar secondary structure contents.

**Figure 3:**
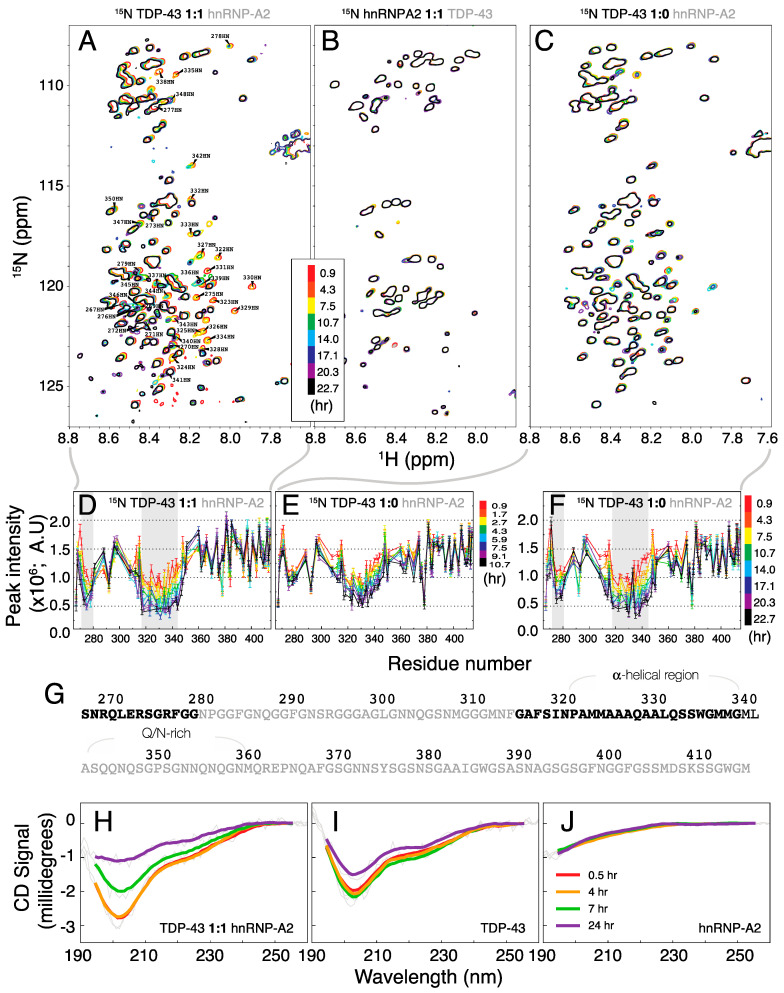
The time evolution of NMR HSQC and circular dichroism spectra indicates that conformational change in TDP-43^266–414^ is accelerated by the presence of hnRNP-A2^288–341^. (**A**–**C**) Time sequences of NMR HSQC spectra of ^15^N-TDP-43 (**A**) in the presence and (**C**) in the absence of hnRNP-A2^288–341^, and (**B**) of ^15^N hnRNP-A2^288–341^ in the presence of TDP-43^266–414^. Assignments are shown for the cross-peaks whose position or intensity clearly change over time. (**D**–**F**) Time sequences of NMR peak intensity profiles as a function of residue number for (**D**) TDP-43^266–414^ mixed with hnRNP-A2^288–341^ and (**E**,**F**) for TDP-43^266–414^ alone. (**G**) Primary sequence of TDP-43^266–414^ with residues highlighted in black if the corresponding NMR intensity decreases over time. The α-helical and Q/N rich regions are also indicated. (**H**–**J**) Circular dichroism spectra at different incubation times of (**H**) the mixture sample, (**I**) TDP-43^266–414^ alone and (**J**) hnRNP-A2^288–341^ only.

Although there were no significant changes initially in the mixture spectra, the intensity of the cross-peaks from residues ~266–277 and ~314–340 in the spectrum of ^15^N-labeled TDP-43^266–414^ (Figure 3A) decreased to the noise level within 9 h in the presence of hnRNP-A2^288–341^ (Figure 3D). No such changes were observed for the mixture of ^15^N-labeled hnRNP-A2^288–341^ with ^14^N-labeled TDP-43^266–414^ (Figure 3B). Many peaks from residues ~340–350 also have distorted shapes (see peak assignments in Figure 3A). The peaks from these residues also decrease over time in the spectra of TDP-43^266–414^ alone, but more slowly (Figure 3C,E). Comparing Figure 3D,F shows that the relative decrease after 9 h in the mixture sample is only observed after 22 h for TDP-43^266–414^ alone. The residues of which the corresponding peaks decrease significantly in intensity are clustered in two regions in the primary sequence (Figure 3G), including the previously identified α-helical region (residues ~314–340) [37,38,39]. Because some of the lost signals originate from the α-helical region, this suggests that the time-dependent transition involves a change in secondary structure. Indeed, in agreement with the NMR data, the CD spectra of the mixture samples vary substantially over time (Figure 3H). This time-dependence is observed to a lesser extent for TDP-43^266–414^ alone (Figure 3I) but not in hnRNP-A2^288–341^ alone (Figure 3J). While changes in the CD spectra of the mixture sample start to appear after 4 h (Figure 3H), the CD spectra of TDP-43^266–414^ vary little over the first 7 h (Figure 3I). The timescales of these changes in secondary structure correspond to those of the decreases in NMR signal.

**Figure 4:**
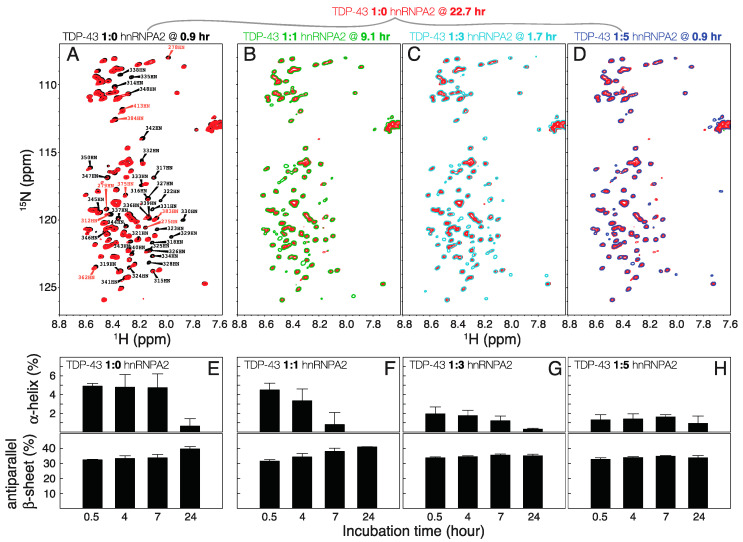
Increasing the amount of hnRNP-A2^288–341^ accelerates transitions from α-helix to β-sheet in TDP-43^266–414^. (**A**–**D**) Comparisons of the NMR HSQC spectrum of TDP-43^266–414^ in the absence of hnRNP-A2^288–341^ after 22 h of incubation (red) with the spectra of (**A**) TDP-43^266–414^ alone after ~1 h incubation (black), (**B**) a 1:1 mixture of TDP-43^266–414^ and hnRNP-A2^288–341^ after ~9 h of incubation, (**C**) a 1:3 mixture of TDP-43^266–414^ and hnRNP-A2^288–341^ after ~3 h of incubation, and (**D**) a 1:5 mixture of TDP-43^266–414^ and hnRNP-A2^288–341^ after less than 1 h of incubation. (**E**–**H**) Proportions of secondary structure elements derived from CD measurements (upper panel: α-helix; lower panel: antiparallel β-sheet) for the same mixtures at different incubation times.

Figure 4A–D show that the more hnRNP-A2^288–341^ is added to TDP-43^266–414^, the faster TDP-43^266–414^ undergoes conformational change. The spectrum of TDP-43^266–414^ alone recorded after 22 h overlaps with those of 1:1, 1:3, and 1:5 mixtures with hnRNP-A2^288–341^ recorded around nine, two and one hours, respectively. The same trends are observed in the time evolutions of the secondary structure contents of the proteins derived from CD measurements (Figure 3H,I and Figure 4E,H). The presence of an equal amount of hnRNP-A2^288–341^ accelerates the decrease of the proportion of α-helix and the increase in the proportion of antiparallel β-sheet. On the contrary, when hnRNP A2^288–341^ is present in three- or five-fold excess, the conformational change is saturated right from the start, with very little α-helix and similar proportions of antiparallel β-sheet at all incubation times (Figure 4G,H, supporting Table S1).

**Figure 5:**
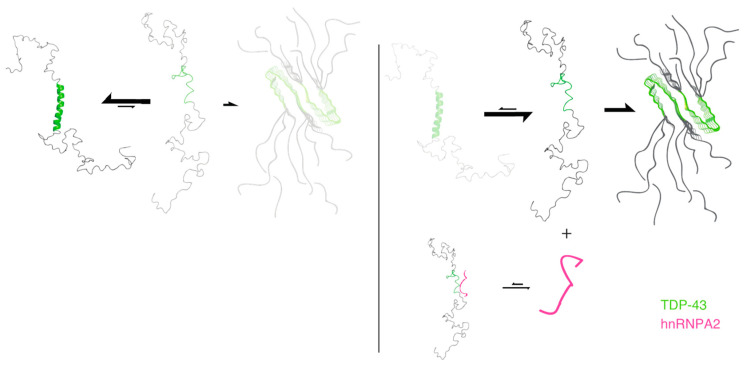
A schematic illustration of the proposed model. In the absence of hnRNP-A2, the IDR of TDP-43 is in equilibrium with both α-helix and coil conformations but tends to aggregate in its coil form (left panel). When the α-helical region of TDP-43′s IDR weakly contacts the IDR of hnRNP-A2 (pink), the coil population of TDP-43 is increased, which enhances its tendency to aggregate (right panel).

The transient and dynamic nature of the α-helical region of TDP-43 is known from previous solution NMR studies [37,39]. Detailed structural information on fibrillized TDP-43 has been obtained by X-ray crystallography for two peptide fragments (residues 321–326 and 333–343) which form an amyloid fibril with antiparallel β-sheets [43]. Cryo-EM images of the amyloid core of TDP-43 covering residues 311–360 are in agreement with the loss of the α-helical component observed here [44]. Although the intrinsic α-to-β transition propensity of TDP-43 has been suggested previously by studies of peptides (residues 311–360) [45,46], the present study demonstrates that this transition is accelerated in the presence of its interacting partner hnRNP-A2 (Figure 5). The monomeric IDR of TDP-43 populates both helix and coil conformations at equilibrium and tends to aggregate in its coil form. When the α-helical region weakly contacts the IDR of hnRNP-A2, the coil population of TDP-43 is increased, which enhances its tendency to aggregate. Although the TDP-43 fibrillization or aggregation could be initiated by many factors, including the truncated RNA-recognition motifs (RRMs) [47], the interaction of the N-terminal domain [48,49], and the level of phosphorylation [50], our study using the IDRs of TDP-43 and hnRNP-A2, demonstrates another factor that also regulates TDP-43 aggregation. Our results may provide clues as to how these two proteins interact in their intact forms, in addition to their RNA-interacting domains.

## 3. Materials and Methods

### 3.1. DNA Constructs

The design of the TDP-43^266–414^ construct has been described previously [37,51]. The cDNA of hnRNP-A2 was provided by Dr. Joseph J-T Huang (Academia Sinica). The cDNA corresponding to residues 288–341 was fused with hexahistidine-tagged SUMO protein at its N-terminal domain to assist purification (His_6_-SUMO-hnRNPA^228–341^) [52]. All constructs were fully sequenced.

### 3.2. Protein Expression and Purification

All proteins were expressed in *Escherichia coli* BL21 (DE3). The protocol used to express TDP-43^266–414^ and hnRNP-A2^288–341^ has been described in detail previously [51,53]. Briefly, overexpressed TDP-43^266–414^ was purified from inclusion bodies dissolved by 8 M urea. An IMAC (immobilized metal-ion affinity chromatography) nickel-charged (Qiagen, Inc., Hilde, Germany) column was used to purify the hexahistidine-tagged protein. The eluted sample then was purified using a C4 reverse phase column (Thermo Scientific, Inc., MA, USA) from an HPLC system. The purified sample was lyophilized for storage. For every experiment, the lyophilized sample was dissolved in 10 mM phosphate buffer at pH 6.5 with protease inhibitor (Roche, Inc., Basel, Switzerland). The protein concentration was determined using the Beer–Lambert law by measuring the absorbance at 280 nm with a NanoDrop UV-VIS spectrometer (Thermo Scientific, Inc.).

In the case of hnRNP-A2^288–341^, the cell lysate supernatant was filtered (0.45 µm) and loaded onto an IMAC column. Ten column volumes (CVs) of 50 mM Tris-HCl with 300 mM NaCl at pH 7.5 were applied to wash the column. The target protein was eluted using five CVs of washing buffer with 500 mM imidazole. A PD-10 column (GE Healthcare, Inc., Chicago, IL, USA) was applied to remove imidazole. A final concentration of 1 mg/mL 6xHis-Ulp1^403–621^ protease was added to the protein solution and left at 4 °C for 2 h to detach the 6xHis-SUMO tag from hnRNP-A2^288–341^. The protease-digested solution was loaded onto a nickel-charged IMAC column and the flow-through was collected. The purified protein was buffer exchanged to 10 mM phosphate buffer at pH 6.5 using a PD-10 column. Protein purity was verified using SDS-PAGE (an example is shown in the Supporting Information, Figure S1. The purified sample was frozen with liquid nitrogen and stored at −80 °C until needed.

### 3.3. NMR Spectroscopy and Data Analysis

^15^N-edited HSQC spectra were recorded using the standard pulse sequence with WATERGATE solvent suppression [54,55]. Peak intensities were measured by fitting the data automatically using non-linear lineshapes [56]. All the HSQC spectra were recorded using a Bruker AVIII 600 MHz spectrometer with a cryogenic probe at 288 K, unless otherwise stated. The data were processed using NMRPipe [56], and peak intensities were obtained using NMRPipe’s non-linear line shape modelling function, including a Lorenz-to-Gauss window function.

### 3.4. Circular Dichroism Spectroscopy

An AVIV model 410 spectropolarimeter was used to collect the circular dichroism spectra. All samples were loaded in a 0.1 mm cuvette. Data were collected and co-added with ten measurements for each data point between 190 nm and 260 nm with an interval of 1 nm. All spectra were recorded at 288 K and the samples were kept in a water bath at 288 K between measurements. All experiments were performed in triplicate. The CD signal was normalized to the sample concentration and the number of residues. The measured theta machine units (*θ*) were converted to Δ*ε* using the following equation [57]:

(1) $$\Delta \varepsilon =\theta \times \frac{0.1\times MRW}{l\times C\times 3298}$$

where *l* is the path length (in cm), *C* is the protein concentration (in mg/mL), and *MRW* is the mean residue weight (molecular weight/residue number, in Daltons). The secondary structure populations were estimated using the program BeStSel [41,42]. All measurements were performed in triplicate.

## 4. Conclusions

Our study demonstrates that the presence of the IDR of hnRNP-A2 accelerates the disease-related α-helix-to-β-sheet transition of the IDR of TDP-43 with residue-specific detail. This is an example of how IDRs affect each other’s behavior and could be more generally applicable to other RBPs. More than 1500 RBPs are present in human cells and most of them have IDRs. In addition to the structured RNA-recognition motifs, our study demonstrates a general mechanism through which RBPs may regulate each other’s functions through IDRs in RBP-mediated gene regulation.

## Supplementary Materials

Supplementary materials can be found at https://www.mdpi.com/1422-0067/21/16/5930/s1. Figure S1: The SDS-PAGE showing different stages of the purification of hnRNP-A2288–341. M: protein size marker; BI/AI: before/after IPTG induction; S/P: supernatant/pellet of the lysed cell; F/W/E: flow-through/wash-through/elution of the first IMAC purification; Bd/Ad: before/after enzyme digestion; Fl2: second flow though the IMAC column. The purified hnRNP-A2288–341 is indicated with the red box. Table S1: The secondary structure population (%) derived from CD measurement using BeStSel.

## Abbreviations

NMR	Nuclear magnetic resonance
IDR	Intrinsically disordered regions
RBP	RNA binding protein
CD	Circular dichroism
TDP-43	Tar DNA binding protein of 43 kDa
hnRNP-A2	Heterogeneous nuclear ribonucleoprotein A2
