# Supplementary material for: Interactions between the Intrinsically Disordered Regions of hnRNP-A2 and TDP-43 Accelerate TDP-43′s Conformational Transition
Source: Int J Mol Sci. 2020 Aug 18;21(16):5930. doi: 10.3390/ijms21165930 (PMC7460674; doi:10.3390/ijms21165930)
Supplement: Supplementary file 1 [file ijms-21-05930-s001.pdf]

International Journal of *Molecular Sciences*

MDPI

Supporting Information

Interactions between the Intrinsically Disordered Regions of hnRNP-A2 and TDP-43 Accelerate TDP-43's Conformational Transition

Wan-Chin Chiang [1], Ming-Hsuan Lee [1], Tsai-Chen Chen [1] and Jie-rong Huang [1,2,3,*]

1 Institute of Biochemistry and Molecular Biology, National Yang-Ming University, No. 155 Section 2, Li-nong Street, Taipei 11221, Taiwan; cmiyc777@gmail.com (W.-C.C.); lkm551200@gmail.com (M.-H.L.); hazelnut.chen.scu@gmail.com (T.-C.C.)

2 Institute of Biomedical Informatics, National Yang-Ming University, No. 155 Section 2, Li-nong Street, Taipei 11221, Taiwan

3 Department of Life Sciences and Institute of Genome Sciences, National Yang-Ming University, No. 155 Section 2, Li-nong Street, Taipei 11221, Taiwan

* Correspondence: jierongh@ym.edu.tw

Received: 28 July 2020; Accepted: 15 August 2020; Published: date

Table S1. The secondary structure population (%) derived from CD measurement using BeStSel.

	Time (h) [a]	1:0 [b]	1:1	1:3	1:5
α [c]	0.5	4.9 ± 0.30	4.5 ± 0.72	1.9 ± 0.74	1.3 ± 0.56
	4	4.8 ± 1.34	3.3 ± 1.26	1.8 ± 0.55	1.4 ± 0.53
	7	4.7 ± 1.47	0.8 ± 1.3	1.2 ± 0.50	1.6 ± 0.26
	10	0.63 ± 0.78	0 ± 0	0.3 ± 0.10	0.9 ± 0.78
β [d]	0.5	32.5 ± 0.38	31.6 ± 1.08	33.9 ± 0.50	32.7 ± 1.13
	4	33.3 ± 1.82	34.4 ± 2.21	34.5 ± 0.66	34.0 ± 0.55
	7	33.9 ± 2.16	38.1 ± 2.10	35.6 ± 0.76	34.9 ± 0.36
	10	39.7 ± 1.59	41.0 ± 0.11	35.2 ± 0.99	33.8 ± 1.48

[a] The incubation time before CD measurements; [b] The TDP-43:hnRNP-A2 ratio; [c] α-helical population (%); [d] Antiparallel β-sheep population.

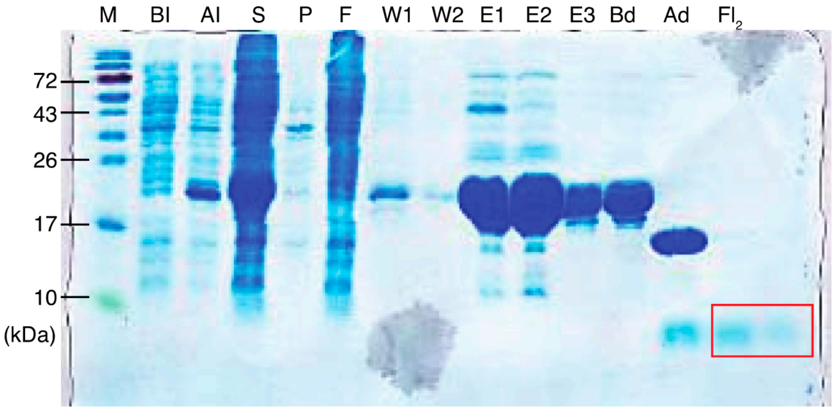

Figure S1. The SDS-PAGE showing different stages of the purification of hnRNP-A2$^{288–341}$. M: protein size marker; BI/AI: before/after IPTG induction; S/P: supernatant/pellet of the lysed cell; F/W/E: flow-through/wash-through/elution of the first IMAC purification; Bd/Ad: before/after enzyme digestion; FI_2: second flow though the IMAC column. The purified hnRNP-A2$^{288–341}$ is indicated with the red box.
